# Supplementary material for: The interactome of intact mitochondria by cross-linking mass spectrometry provides evidence for coexisting respiratory supercomplexes
Source: Mol Cell Proteomics. 2017 Dec 8;17(2):216–32. doi: 10.1074/mcp.RA117.000470 (PMC5795388; doi:10.1074/mcp.RA117.000470)
Supplement: Supplemental Data [file supp_RA117.000470_133922_0_supp_23978_fzffwf.zip › spectra_annotation/mito_spectra_annotation/77-1-8-1-2-1.pdf]

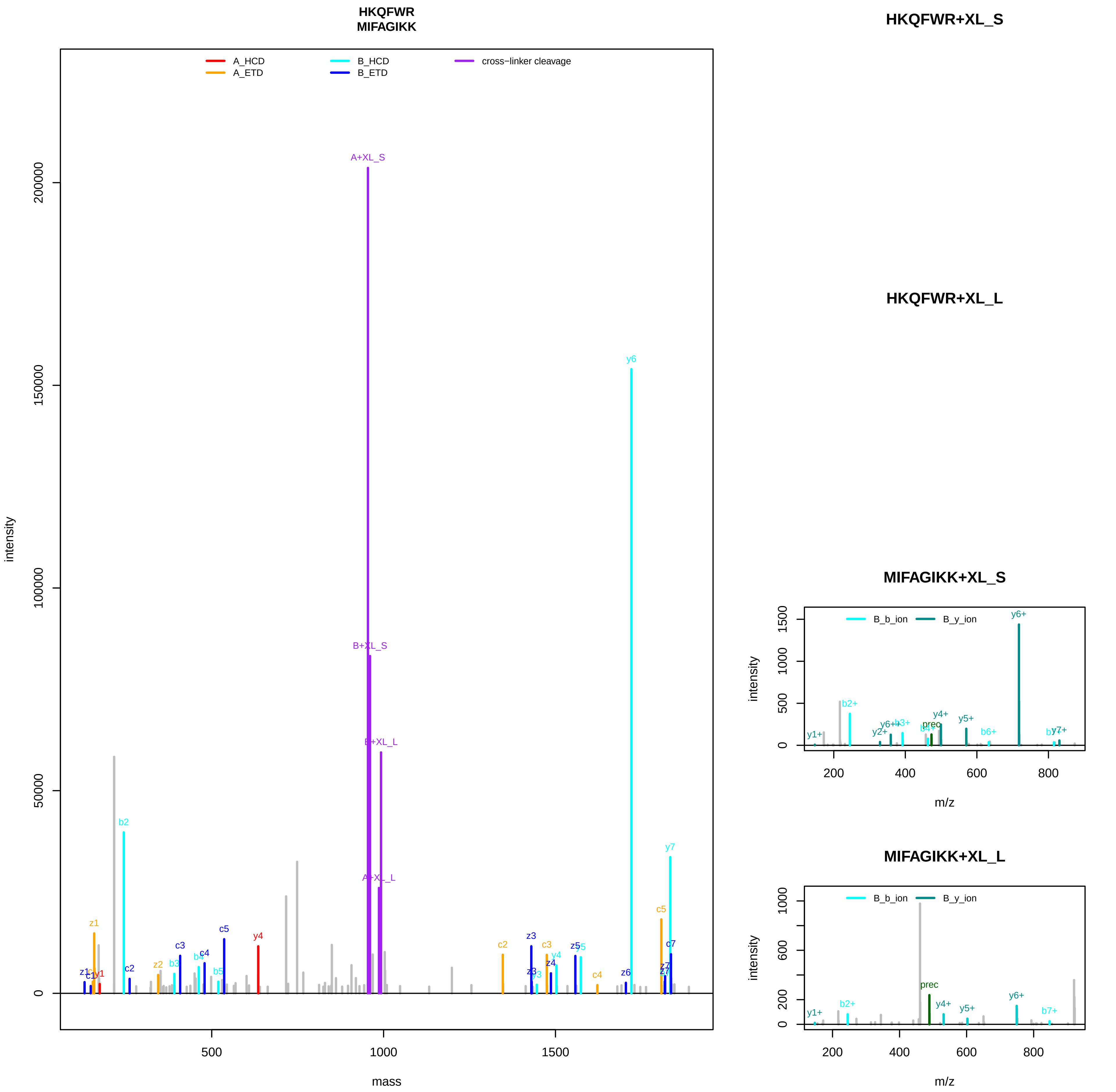

HKQFWR
MIFAGIKK
A_HCD
A_ETD
B_HCD
B_ETD
cross−linker cleavage
A+XL_S
B+XL_S
B+XL_L
A+XL_L
y6
y7
c5
c7
z7
z6
c4
z5
y5
z4
z3
y3
c3
y4
c2
b2
z1
c1
y1
c2
z2
b3
c3
b4
c4
b5
c5
intensity
mass
0
50000
100000
150000
200000
500
1000
1500
HKQFWR+XL_S
HKQFWR+XL_L
MIFAGIKK+XL_S
B_b_ion
B_y_ion
y6+
y1+
b2+
y2+
y6++
b3+
b4+
prec
y4+
y5+
b6+
b7+
y7+
intensity
m/z
0
500
1000
1500
200
400
600
800
MIFAGIKK+XL_L
B_b_ion
B_y_ion
prec
y1+
b2+
y4+
y5+
y6+
b7+
intensity
m/z
0
200
600
1000
200
400
600
800
